# Economic burden of stroke attributable to excess body mass in Hungary: a population-attributable fraction analysis

**DOI:** 10.1186/s12889-026-26355-y

**Published:** 2026-02-03

**Authors:** Tamas Jarecsny, Csilla Arvane Egri, Roland Kosik, Richard Schwab, Laszlo Mechtler, Gergo Jozsef Szollosi, Laszlo Schandl, Gyula Tomasics, Istvan Gyuricsko, Eszter Melinda Pazmandi, Ferenc Fazekas, Monika Fekete

**Affiliations:** 1 Department of Neurology and Stroke, Saint John’s Central Hospital of North Buda, Budapest, Hungary; 2 Doctoral College of Semmelweis University, Budapest, Hungary; 3 National Public Health and Pharmaceutical Center, Budapest, Hungary; 4 BiomTeam@ MiND Brain-Gut Center, Budapest, Hungary; 5 DENT Neurologic Institute, Buffalo, NY USA; 6 Faculty of Economics and Business, University of Debrecen, Debrecen, Hungary; 7 Department of Internal Medicine-Diabetology, Saint John’s Central Hospital of North Buda, Budapest, Hungary; 8 Faculty of Medicine, Semmelweis University, Budapest, Hungary; 9 Semmelweis University Andras Peto Faculty, Budapest, Hungary; 10 Health Services Management Training Centre, Semmelweis University, Budapest, Hungary; 11 Institute of Preventive Medicine and Public Health Faculty of Medicine, Semmelweis University, Budapest, Hungary; 12 Fodor Center for Prevention and Healthy Aging, Semmelweis University, Budapest, Hungary; 13 Health Sciences Division, Doctoral College, Semmelweis University, Budapest, Hungary

**Keywords:** Body mass index, Stroke, Population attributable fraction, Cost of illness, Hungary, Economic burden

## Abstract

**Background:**

The prevalence of obesity in Hungary ranks among the highest in the European Union, representing a significant healthcare burden. In modern medicine, stroke remains one of the major cardiovascular cost drivers, with obesity being a key modifiable risk factor.

**Aims:**

To estimate annual stroke-related healthcare cost savings achievable through population-level reduction in body mass index (BMI) in Hungary, based on five international risk models.

**Methods:**

A cross-sectional study of 2,442 adults was conducted between June and August 2022 via the nationwide “Bringing Screening to You” program. Post-stratification weighting by sex and age was applied to align with the 2022 national census. Population-attributable fraction (PAF) analysis incorporated five published BMI–stroke models (HUNT, CHARLS-male cohort, Physicians’ Health Study, meta-analysis, INTERSTROKE) and was applied to the €1.016 billion national stroke cost for 2022. Uncertainty was assessed using a 10,000-iteration Monte Carlo simulation.

**Results:**

Weighted overweight and obesity prevalence was 72.5% (95% CI: 70.6–74.4%), one of the highest observed among Central and Eastern European countries. The PAF of stroke cases associated with excess BMI ranged from 9.7% to 20.4%, corresponding to annual cost savings of €99–207 million under a theoretical maximum scenario of complete BMI normalization, assuming proportional cost reduction. Conservative estimates (HUNT model) projected €99 million in savings (95% CI: 75–127), while higher estimates (Physicians’ Health Study) yielded €207 million (95% CI: 145–265).

**Conclusions:**

Population-level BMI reduction could reduce Hungary’s annual stroke related healthcare expenditure by at least €99 million, representing substantial potential savings. These findings support implementation of population-level comprehensive obesity prevention strategies.

**Supplementary Information:**

The online version contains supplementary material available at 10.1186/s12889-026-26355-y.

## Introduction

Overweight and obesity have become health-economic emergencies worldwide [1–4]. In the European region, the prevalence of overweight and obesity has more than doubled between 1975 and 2016, hence rising from 10% to 23% [5]. As of 2022, more than 2.5 billion adults were overweight while 890 million were obese globally—representing approximately 16% of the world’s population [6]. If current trends continue, the World Obesity Federation projects that by 2035 more than 4 billion people. or 51% of humanity, will live with excess body mass, generating an annual economic burden approaching $4.32 trillion [6]. Excess adiposity drives costs across virtually every major disease group, yet cardiovascular conditions—and stroke in particular—account for a disproportionate share of these expenditures [7–17].

Beyond its direct role as a stroke risk factor, obesity functions as a critical conduit for multiple secondary comorbid conditions that independently elevate stroke risk [18]. Patients with severe obesity (BMI classes II-III) exhibit substantially elevated morbidity and mortality [19]. Obesity is a recognized risk factor for cardiovascular disease, type 2 diabetes mellitus, malignancy, asthma, obstructive sleep apnea, non-alcoholic fatty liver disease, and gallbladder diseases [19–23]. Consequently, the obesity epidemic is accompanied by escalating prevalence of these obesity-related comorbidities [19–24]. Obesity clusters with other stroke risk factors [25]. This synergistic pattern substantially amplifies stroke incidence beyond what obesity alone would predict [26–29].

Stroke imposes considerable economic burden globally, encompassing direct healthcare costs, productivity losses, and long-term care requirements [30–32]. The American Heart Association projects that stroke-related expenditures will rise by 535% between 2020 and 2050, reaching $1.490 trillion annually in the United States [8, 31]. Similar patterns are emerging in Europe, where stroke already accounted for €60 billion in 2017 across 32 countries, of which €27 billion were direct healthcare costs [10]. Rising costs reflect both population aging and the ballooning prevalence of modifiable risk factors such as obesity, hypertension, and hyperglycemia [33–35].

Within the European context, Hungary illustrates these dynamics with unusual clarity. National survey data showed one of the highest combined overweight-and-obesity prevalence in the European Union—72.5% versus the EU mean of 51.6% [36]. Stroke mirrors this excess risk: annual Hungarian stroke expenditures were estimated at €1.02 billion in 2022, translating to €105 per capita—representing an 81% increase from the 2017 estimate of €58 per capita [10, 37]. Despite these mounting costs, evidence quantifying the specific proportion of Hungary’s stroke burden attributable to excess body mass remains limited. In order to address this knowledge gap, it is essential to estimate how much of the national stroke burden could theoretically be prevented through population-level weight reduction.

## Aims

This study therefore estimates the annual stroke-related costs attributable to excess body mass in Hungary using population-attributable fraction (PAF) methodology and five independent international risk estimation models. By translating relative risks and national cost data into monetary terms, this analysis provides quantitative evidence for the potential economic returns achievable through effective BMI-reduction strategies—information urgently needed by clinicians, health-service planners, and policy-makers.

## Method

### Study design and participants

The data for this study were collected as part of the **“**Helybe visszük a szűrővizsgálatokat**”** program (literally *“Bringing Screening to You”*), an ongoing nationwide mobile health screening initiative launched by the National Public Health Center in 2019 and overseen since 2023 by the National Hospital Directorate (OKFŐ). The program provides free, on-site preventive medical screenings to improve health equity, particularly in underserved areas. Services include cardiovascular risk assessments (blood pressure, blood sugar, cholesterol checks, ECG), oral and skin cancer screenings, gynecological exams, as well as general health evaluations like body composition measurements and lung function tests [38].

We conducted a cross-sectional, observational study between June 1 and August 31, 2022, within the framework of this program. A map of all screening site locations and their contribution to the study population is provided in Supplementary Fig. 1. Of the 5,059 volunteers across 108 Hungarian municipalities, 2,617 were excluded due to missing height or weight data or age below 18 years. The high exclusion rate reflects incomplete participation in specific screening component rather than systematic exclusion by demographic criteria: volunteers could choose which screening modules to attend (e.g., attending cardiovascular assessment but nod body composition measurement). The final analytical sample comprised 2,442 participants with complete anthropometric data: 1,737 women (71.1%) and 705 men (28.9%), with a mean age of 52.9 years (SD ± 14.5; range 18–87) (Table 1). To correct for sex- and age-related sampling imbalances, post-stratification weighting was applied (see Sect. 3.4). Sex distribution did not differ significantly between included and excluded participants (*p* = 0.053), whereas age did (*p* = 0.007).

**Table 1:** Key demographic characteristics of included vs. excluded participants

Characteristic	Included sample (*n* = 2,442)	Excluded sample (*n* = 2,617)	Between-group comparison
Mean age (years)	52.9 ± 14.5	51.2 ± 18.7	t = 2.70; *p* = 0.007
Median age (years)	54 (IQR 42–64)	52 (IQR 38–64)	—
Age range (years)	18–87	1–87	—
Sex distribution – Women	1,737 (71.1%)	1751 (66.9%)	χ²(1) = 3.77; *p* = 0.053
Sex distribution – Men	705 (28.9%)	866 (33.1%)	—
Mean BMI (kg/m²)	28.6 ± 6.0	27.7 ± 6.1	—
Median BMI (kg/m²)	28.0 (IQR 24.4–32.1)	27.1 (IQR 23.5–31.4)	—
BMI range (kg/m²)	14.7–73.9	15.4–52.9	—

*Abbreviations: n* Number of participants, *BMI* Body mass index, *IQR* Interquartile range, *χ²* Chi-square test, *t* Student’s t-test, *p* p-value

### Data collection

Anthropometric measurements were performed using an InBody 270 bioelectrical impedance analyzer (body weight accuracy ± 0.1 kg) and a Soehnle ultrasonic stadiometer (height accuracy ± 0.5 cm). All measurements were taken with participants wearing light clothing and no shoes, preferably in the morning at least two hours after eating. Body mass index was calculated as weight (kg) divided by height squared (m²). BMI values were recorded to one decimal place, and heights were rounded to the nearest whole centimeter. BMI categories followed World Health Organization standard classifications: underweight (< 18.5), normal weight (18.5–24.9), overweight (25.0–29.9), and obesity (≥ 30.0 kg/m²).

### Missing data and data quality

No missing BMI values occurred in the final dataset. All recorded measurements fell within physiologically plausible ranges, and therefore no observations were excluded from the analysis.

### Weighting strategy

To address potential selection bias from voluntary participation, post-stratification weights (Table 2) were applied to align the sample distribution with the 2022 Hungarian census. Post-stratification weighting was applied only to age and sex for three reasons: [1] age showed significant selection bias (mean age 52.9 vs. 51.2 years, *p* = 0.007), while sex distribution was marginally significant (*p* = 0.053), necessitating correction for these primary demographic variables; [2] including additional demographic variables would create sparse cell problems and generate unstable weight estimates with inflated confidence intervals; and [3] sensitivity analyses confirmed robustness, alternative weighting strategies (sex-only, age-only, combined, and exclusion of extreme BMI values) altered BMI prevalence by less than 0.2% points.

**Table 2:** Post-stratification weighting factors

Dimension	Census (%)	Sample (%)	Weight
Women	52.0	68.1	0.764
Men	48.0	31.9	1.505
Age 18–44 yrs	33.0	28.4	1.162
Age 45–64 yrs	33.0	46.7	0.707
Age ≥ 65 yrs	20.8	24.9	0.835

*yrs* Years

Each participant’s combined weight was calculated as:

$$W_{i}=W_{sex} \times W_{age group}$$

### Population-attributable fraction analysis

The population-attributable fraction (PAF) quantifies the proportion of disease cases that could theoretically be prevented if a specific risk factor were eliminated, assuming a causal relationship between exposure and outcome. To estimate the fraction of stroke cases attributable to excess body mass, we applied the standard PAF formula:

$$\:PAF=\sum\:_{i=1}^{n}\:{p}_{i}\cdot\:\frac{(R{R}_{i}-1)}{R{R}_{i}}$$

where $\:{p}_{i}$ represents the proportion of the population in BMI category $\:i$, and $\:R{R}_{i}$ represents the relative risk for stroke in that category compared to normal weight (18.5–24.9 kg/m²).

No domestic BMI-stroke risk model has been previously developed or validated within the Hungarian population. Consequently, we applied five published international risk models selected to encompass a broad range of epidemiological contexts and methodological approaches. These models were chosen according to three criteria: [1] methodological rigor and outcome validation [2], relevance to Central and Eastern European healthcare systems where applicable, and [3] global diversity to capture the spectrum of plausible BMI-stroke associations. The resulting estimates therefore span from conservative scenarios based on Nordic and other European-type setting to more exploratory scenarios derived from non-Western populations and non-linear risk functions, providing a comprehensive range of estimates for Hungarian policy consideration:

- HUNT Study (Norway) [39]: Prospective cohort (*n* = 14,139; 12-year follow-up) demonstrating approximately 30% increased ischemic stroke risk among overweight and obese participants. Stroke events were hospital-confirmed via linkage to the Norwegian Patient Registry, providing high diagnostic validity. This model offers conservative estimates from a Nordic population with healthcare system characteristics similar to Hungary.
- CHARLS male cohort (China) [40]: Longitudinal study of men aged 45 + years revealing a non-linear BMI–stroke association, with risk peaking at BMI ~ 26 kg/m². Stroke was ascertained through self-report with physician confirmation. Despite geographic and dietary differences, this model demonstrates BMI–stroke relationships in high-prevalence obesity settings.
- Physicians’ Health Study (USA) [41]: Prospective cohort of 21,867 male physicians demonstrating that obesity (BMI ≥ 30) nearly doubled stroke risk compared with normal BMI (RR 1.91, 95% CI 1.45–2.52). Outcomes were validated through medical record review, ensuring high-quality endpoint ascertainment. This model represents western dietary patterns and lifestyle factors increasingly prevalent in Hungary.
- Meta-analysis (international data) [42]: Systematic review and meta-analysis synthesizing 24 prospective cohorts encompassing approximately 5.8 million participants. This model demonstrates dose-response increases in stroke risk with rising BMI, with stronger associations observed among men and for ischemic stroke subtypes. The pooled international data provide the most comprehensive risk estimates across diverse populations
- INTERSTROKE (32 countries) [43]: Global case-control study conducted across 32 countries including Central and Eastern European populations. This study demonstrated that both general obesity (BMI) and central obesity (waist-to-hip ratio) significantly increase stroke risk (OR 1.65, 95% CI 1.36–1.99 for highest vs. lowest BMI tertile). The geographic diversity and inclusion of middle-income countries make this model particularly relevant for Hungarian population risk estimation.

### Cost estimation

We applied calculated PAF values to Hungary’s 2022 stroke-related healthcare expenditure of €1.016 billion, derived from national health insurance data. In doing so, we assumed that a proportional reduction in stroke incidence would lead to a linear reduction in total stroke-related healthcare costs. Under this assumption, annual cost savings were estimated by multiplying each model’s PAF by the total national stroke costs. This approach implicitly assumes that averted stroke events have, on average, the same cost profile as all stroke cases and that stroke care expenditures scale approximately linearly with changes in case volume. Complete model parameters are provided in Supplementary Materials Appendix C.

### Statistical analysis

All analyses were performed in R version 4.2.1 (The R Foundation for Statistical Computing, Vienna, Austria) using the tidyverse, survey, epiR, and boot packages. Descriptive statistics for continuous variables were presented as mean ± standard deviation and median (interquartile range), and categorical variables as relative frequencies.

Weighted prevalence estimates were derived using both Taylor linearization and bootstrap resampling techniques (B = 1,000 replications), with 95% confidence intervals. Differences in BMI category distributions between sexes were assessed by Pearson’s chi-square test (χ²), and effect size quantified using Cohen’s w. The association between age groups and BMI categories was measured with Cramér’s V.

Sensitivity analyses explored four weighting strategies: sex-only weighting, age-group-only weighting, combined sex + age-group weighting, and exclusion of extreme BMI values (< 15 or > 60 kg/m²), which altered prevalence estimates by less than 0.2% points, confirming the robustness of the main results.

Uncertainty in the PAF estimated was quantified by using a 10,000-iteration Monte Carlo simulation to generate confidence intervals, incorporating stochastic variation in post-stratification weights, BMI category prevalences, and model-specific relative risks.

An a priori power analysis was conducted to ensure adequate statistical precision for detecting meaningful differences in BMI distribution across the four World Health Organization categories (underweight, normal weight, overweight, obese). Using standard power calculation formulas for chi-square goodness-of-fit tests, we specified an effect size of Cohen’s w = 0.20 (small-to-medium effect, corresponding to a meaningful difference in BMI category proportions), an alpha level of 0.05 (two-tailed), and a desired statistical power of 0.80 (i.e., 80% probability of detecting a true effect if it exists). Under these parameters, the minimum required sample size was calculated as *n* = 1,964. Our actual sample of *n* = 2,442 exceeded this threshold, providing approximately 90–95% statistical power, sufficient to estimate BMI prevalences within each WHO category within narrow confidence intervals (< 5% points) and to ensure robust weighted estimates across sensitivity analyses.

Detailed statistical methods are described in Supplementary Materials Appendix A.

## Results

### Sample characteristics

After the post-stratification weighted analysis, the sample closely reflected the demographics of the Hungarian adult population. Mean BMI was 29.7 ± 6.4 kg/m², with a median of 28.9 kg/m² (IQR: 25.1–33.6) and values ranging from 15.4 to 59.8 kg/m².

### BMI distribution and prevalence

The weighted prevalence analysis showed that 2.3% (95% CI: 1.7–3.0%) of participants were classified as underweight, while 27.0% (95% CI: 25.2–28.9%) had a normal body mass index. Overweight individuals accounted for 30.5% (95% CI: 28.6–32.4%) of the population, and 40.3% (95% CI: 38.3–42.3%) were categorized as obese. Taken together, the combined prevalence of overweight and obesity reached 72.5% (95% CI: 70.6–74.4%), highlighting the exceptionally high burden of excess body weight among Hungarian adults.

### Population-attributable fraction results

Applying five independent BMI–stroke risk models yielded broadly consistent estimates with the potential reduction in stroke burden associated with population-level BMI improvements. The PAF ranged from 9.7% in the HUNT Study to 20.4% in the Physicians’ Health Study, with intermediate values for CHARLS (16.3%), INTERSTROKE (18.7%) and the meta-analytic model (20.1%) (Table 3). These PAF values indicate that under a complete BMI normalization scenario (all adults achieving normal weight [18.5–24.9 kg/m^2^]), the corresponding proportion of annual stroke cases could theoretically be prevented. In absolute monetary terms, applying these PAF estimates to the 2022 national stroke cost (€1.016 billion) yielded potential annual healthcare savings ranging from €99 million (95% CI: €75–127 million) to €207 million (95% CI: €145–265 million). INTERSTROKE produced estimates near the upper mid-range (savings €190 million, 95% CI: €162–218 million), while CHARLS (male cohort) suggested a moderate-to-high effect (savings €166 million, 95% CI: €83–242 million). The meta-analysis yielded a similar central estimate (PAF of stroke cases associated with excess BMI 20.1%), but with the widest uncertainty bounds (95% CI: 3.4–35.6%; savings €204 million, 95% CI: €35–362 million), reflecting between-study heterogeneity. Across models, summary statistics indicate a mean PAF value of 17.0% (± 3.9%) and mean annual savings of €173 million (±€40 million), implying that roughly one-sixth of current stroke expenditure could be avoided under the modeled BMI shift. Importantly, despite methodological and population differences among models (cohort vs. case-control designs; sex-specific vs. mixed-sex samples; ischemic-only vs. total stroke outcomes), the central tendency converges on a 10–20% PAF value range.

**Table 3:** Population-attributable fraction of stroke cases associated with excess BMI and estimated annual stroke-related cost savings by model

Model	PAF (%)	95% CI	Annual stroke cost savings (€M)	95% CI
HUNT Study	9.7	7.4–12.5	99	75–127
CHARLS (male)	16.3	8.2–23.8	166	83–242
INTERSTROKE	18.7	15.9–21.5	190	162–218
Meta-analysis	20.1	3.4–35.6	204	35–362
Physicians’ Health	20.4	14.3–26.1	207	145–265

*Abbreviations: PAF* Population-attributable fraction, *CI* Confidence interval, *€* Euro, *€M* Million euros, *HUNT Study* The Nord-Trøndelag Health Study,* CHARLS* China Health and Retirement Longitudinal Study, *INTERSTROKE* International Study of Risk Factors for Stroke; Physicians' Health, Physicians' Health Study

## Discussion

### Principal findings

This nationwide analysis of 2,442 adults participating in the “Bringing Screening to You” program revealed a 72.5% prevalence of excess body weight in Hungary after post-stratification weighting for age and sex. To contextualize our findings, we compared our measured 2022 data with 2019 international prevalence estimates: measured data from OECD and WHO indicated approximately 68% combined overweight and obesity 68% [44, 45], while self-reported Eurostat data from 2019 showed 64.1% combined overweight/obesity prevalence, with EU mean of 51.6% [36]. The consistent 5–10% point gap between measured and self-reported estimates in European populations suggests our measured 72.5% in 2022 reflects both true epidemiological changes and the established methodological difference between measurement approaches [46, 47].

Furthermore, significant sex differences emerged, with unweighted prevalence of 63.9% in men versus 77.2% in women. This pattern aligns with international data showing higher obesity prevalence among women in high-income countries, potentially reflecting hormonal factors, cultural dietary patterns, and differing physical activity levels [48]. Post-stratification weighting altered prevalence by only 0.4–1.4% points, confirming the robustness of our weighting approach.

### International comparisons and regional analysis

In regional comparison, Hungary’s prevalence of overweight and obesity stands relatively higher than that of neighboring Central and Eastern European countries (see in Appendix D) [49, 50]. Slovakia reports a combined prevalence of 59.2% regarding overweight and obesity, while Romania shows 54.7% [51]. This 13–18% point gap difference suggests that Hungary faces unique challenges, potentially related to specific dietary customs, socioeconomic transitions, and healthcare system constraints characteristic of post-socialist countries [52–55].

The 20.9% point excess above the EU average (51.6%) represents a 40.5% relative increase, highlighting the urgent need for targeted interventions. The rapid 8.4-percentage point increase between 2019 and 2022 may reflect COVID-19 pandemic effects, including reduced physical activity, modified eating patterns, and healthcare system disruptions affecting preventive care [56–59].

Recent epidemiological surveys of stroke and its risk factors within diverse populations demonstrate geographic heterogeneity in prevalence patterns [60]. The Hungarian population exhibits particularly elevated prevalence of multiple concurrent stroke risk factors: obesity (72.5% versus 51.6% EU mean), hypertension (35.2%), dyslipidemia (55.2%), and smoking (29.6%) [61]. These clustering patterns establish that excess body mass represents a common and modifiable component of stroke risk within Hungary.

### Economic modeling and policy implications

Applying five international BMI–stroke risk models, the PAF of stroke cases attributable to excess BMI ranged from 9.7% (95% CI: 7.4–12.5%) (HUNT Study) to 20.4% (95% CI: 14.3–26.1%) (Physicians’ Health Study). When applied to the €1.016 billion annual national stroke expenditure, these PAF estimates translate to potential annual cost savings ranging from €99 million to €207 million, averaging €173 million (SD: €40 million), under a complete BMI normalization scenario—representing a theoretical maximum rather than a realistic policy outcome.

Our estimates align with published models: Wang et al.’s meta-analysis model (8.7% RR reduction; €89 M savings) [42], INTERSTROKE (18.7%; €190 M) [43], and CHARLS male cohort [40]. With Hungary’s 2022 stroke economic burden at €1.016 billion [37], these savings underscore substantial preventive potential.

Evidence-based interventions demonstrate substantial returns: fiscal policies (sugar-sweetened beverage taxes) achieve 0.3–0.8 kg/m² BMI reduction [62–64], school-based programs show $6–55 return per $1 invested [65], and Mediterranean diet interventions yield cost-effectiveness ratios of -€626 per QALY [66–72]. Worksite wellness programs covering 15–20% of employees produced 1.8–2.1 kg/m² BMI reductions [73–76]. These evidence-based interventions could yield substantial returns on public health investment.

### Digital health and internet of things-enabled prevention as implementation tools

Translating these estimates into effective prevention requires feasible implementation strategies. Digital health technologies and Internet of Things (IoT)–enabled solutions—such as wearable activity trackers, connected weight and body composition scales, and digital dietary monitoring platforms—represent promising tools that can assist obesity prevention and long-term control of lifestyle-related stroke risk factors [77–82].

Evidence from randomized controlled trials and meta-analyses indicates that such tools are associated with modest but significant and sustained improvements in physical activity levels and body weight when embedded within structured lifestyle interventions [83], typically reducing body weight by 1–2 kg over 6–12 months compared with standard care alone [79].

In the Hungarian healthcare context, where obesity prevalence is among the highest in Europe and access to sustained clinical follow-up remains limited in rural and underserved areas [72, 76, 84, 85], IoT-enabled home monitoring could help bridge the gaps in clinical follow-up and enable early identification of individuals at highest stroke risk [81, 86]. When integrated into existing prevention frameworks, digital monitoring technologies can thus complement established clinical and policy-based strategies aimed at reducing stroke burden [87, 88].

### Limitations, future research, and implementation considerations

The cross-sectional design and volunteer-based recruitment may introduce selection bias, though post-stratification weighting addressed major demographic imbalances in age and sex. However, weighting could not extend to region, socioeconomic status, or educational attainment because multivariate stratification would have created sparse cells and generated unstable weight estimates with inflated confidence intervals. As a result, the representativeness of the sample with respect to geographic, socioeconomic, and educational subgroups remains uncertain, and overweight/obesity prevalence may be overestimated in more deprived strata and underestimated in more advantaged groups.

International BMI-stroke models may not fully capture Hungarian population characteristics, highlighting the need for domestic longitudinal studies. Geographic coverage, while extensive across 108 municipalities, may not represent all socioeconomic strata equally. Sensitivity analyses are presented in Supplementary Materials Appendix B.

A further limitation concerns the cost modelling approach. Annual stroke-related healthcare expenditure was assumed to scale linearly with changes in stroke incidence; that is, we applied model specific PAF values directly to aggregate national stroke costs. This simplifying assumption was necessary because detailed Hungarian cost-of-illness data disaggregated by stroke subtype, severity, care pathway, and recurrence status are not currently available. In reality, the marginal cost of additional stroke cases may differ from the average cost (e.g., due to fixed infrastructure costs, case-mix differences, or threshold effects in long-term care utilization), implying that true savings under BMI reduction scenarios could be somewhat higher or lower than our linear estimates.

Establishing a national stroke registry remains essential for validating international model applications and quantifying actual stroke incidence patterns. Prospective cohort studies tracking BMI, obesity trends, lifestyle factors, and health outcomes would enable more precise risk estimation and targeted interventions.

Implementation requires addressing Central and Eastern European-specific challenges. The dual burden of physical inactivity (59% of adults) and obesity (72.5%) necessitates comprehensive, multi-sector approaches [89]. Future cost-effectiveness analyses should incorporate QALY-based evaluations, productivity impacts, and informal caregiving costs to capture full societal benefits of BMI-reduction strategies. The substantial economic potential demonstrated—minimum €99 million annual savings—provides compelling evidence for urgent policy action and sustained investment in population health interventions.

## Conclusions

Hungary faces an extraordinary obesity burden, with 72.5% of adults classified as overweight or obese—the highest prevalence in Central and Eastern Europe. Under a theoretical maximum scenario of complete BMI normalization and proportional cost scaling, population-level BMI normalization could theoretically avert between 9.7% and 20.4% of Hungary’s annual stroke burden, potentially reducing stroke-related healthcare expenditure by at least €99 million, with upper estimates reaching €207 million annually. These findings provide compelling economic evidence for the urgent implementation of comprehensive obesity prevention strategies. National BMI-reduction strategies could cut stroke spending by up to 20%, freeing approximately €200 million annually for other public health priorities.

Given projected increases in stroke-related costs over the coming decades, the economic case for population-level obesity prevention will only strengthen. Hungary’s findings demonstrate that even modest improvements in population BMI distribution could yield substantial healthcare savings while improving population health outcomes.

## Supplementary Information

Supplementary Material 1

## Data Availability

The datasets used and/or analysed during the current study are available from the corresponding author on reasonable request.
