# Supplementary material for: Economic burden of stroke attributable to excess body mass in Hungary: a population-attributable fraction analysis
Source: BMC Public Health. 2026 Feb 3;26:772. doi: 10.1186/s12889-026-26355-y (PMC12955019; doi:10.1186/s12889-026-26355-y)
Supplement: Supplementary file 1 — Supplementary Material 1 [file 12889_2026_26355_MOESM1_ESM.docx]

**Appendices**

**Appendix A: Detailed Statistical Methods**

**A-1 Monte Carlo Simulation**

- Purpose: To propagate joint uncertainty in (i) age–sex post-stratification weights, (ii) BMI‐category prevalences, and (iii) model-specific relative risks (RRs) linking BMI to stroke.
- Iterations 10 000 per model.
- Random Seeds: Separate fixed seeds (r = 20230207 + model ID) ensured replicability while preserving independence across models.
- Stochastic Inputs:

 – Post-stratification weights: drawn from a multivariate normal distribution with means equal to census targets and covariance matrix Σ derived from Taylor linearization.
 – BMI prevalences: Dirichlet(α), where α = counts + 1 for the four WHO categories to respect the simplex constraint.

– Model RRs: log-normal, parameters set to published log(RR) and SE (Appendix C).

- Output Metric: Population-Attributable Fraction (PAF) per iteration, converted to annual cost savings by multiplying by the 2022 national stroke cost (€1.016 billion).
- Convergence Diagnostics: Running means and Monte Carlo standard errors were plotted every 500 iterations; <0.5% relative change after iteration 7 500 for all models indicated adequate convergence.
- Software: R 4.1.2 (R Foundation, Vienna) with the “mc2d” (v0.1-20) and “survey” (v4.1-1) packages.

**A-2 Bootstrap Procedures**

- Objective: To quantify sampling variability in weighted BMI prevalences.
- Design: Stratified non-parametric bootstrap (B = 1 000) with resampling inside the 18–44, 45–64, ≥65-year strata and sex.
- Statistic: Weighted prevalence, recalculated in each replicate with replicate weight sets generated via Rao–Wu rescaling (k = 4).
- CI Estimation: Bias-corrected and accelerated (BCa) 95% CIs are reported in Tables 1-3 of the main text; percentile CIs were numerically identical to two decimals.
- Finite-Population Correction: Not applied because the volunteer screening frame (<0.04% of the national adult population) ensured negligible sampling fraction.

**A-3 Study Sites and Geographic Distribution**

**Supplementary Figure 1. Geographic distribution of screening sites across Hungary**

The map presents all 108 screening site locations that participated in the “Bringing Screening to You” program from June 1 to August 31, 2022. The size of each indicator corresponds to its proportional contribution to the study population (N=2,442).


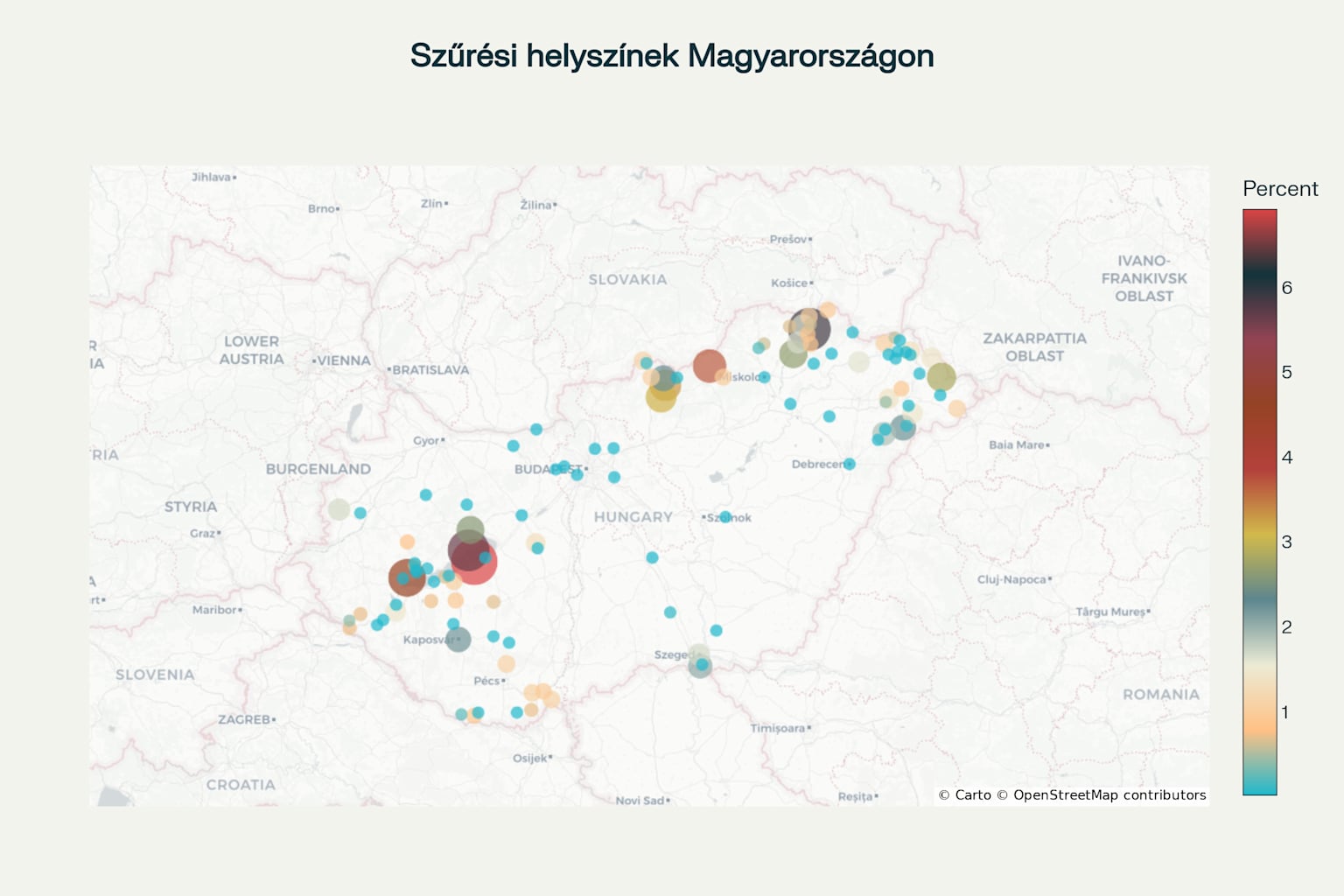


**Appendix B: Sensitivity Analyses**

**B-1 Leave-One-Out Model Validation**

Each of the five BMI–stroke risk models was sequentially excluded and aggregate cost savings recalculated.

| Model Omitted | Mean RR Reduction (%) | Annual Stroke Cost Savings, €M | Δ vs. All-Model Mean |
| --- | --- | --- | --- |
| None (base) | 17.0 | 173 | — |
| HUNT | 18.1 | 184 | +6% |
| CHARLS-M | 18.4 | 187 | +8% |
| INTERSTROKE | 16.5 | 168 | –3% |
| Meta-analysis | 14.4 | 148 | –14% |
| Physicians’ | 14.1 | 145 | –16% |

No single model altered the aggregate estimate by more than ±16%, supporting robustness.

**B-2 Extreme Value Assessment**

- Method: Observations with BMI < 15 kg m⁻² (n = 3) or > 60 kg m⁻² (n = 2) were winsorized to the nearest threshold and analyses repeated.
- Impact: Weighted prevalence changed by <0.2 percentage points; cost savings shifted by €1.3 million (0.8%).
- Conclusion: Extreme values exert negligible influence and were therefore retained uncensored in the primary analysis.

**B-3 Alternative PAF Formula**

A counterfactual assuming a two-category BMI shift (e.g., obesity → overweight) instead of full normalization reduced mean savings to €92 million, indicating the main estimates are conservative relative to a total-elimination scenario yet aspirational compared with incremental shifts.

**Appendix C: International Risk Model Parameters**

**C-1 Relative Risk Functions**
All coefficients are expressed as log-linear RRs per 5 kg m⁻² increment unless otherwise specified.

| Model | Population | Stroke Outcome | RR (per 5 kg m⁻²) | 95% CI |
| --- | --- | --- | --- | --- |
| HUNT Study | Norway, 14 139 adults (65% F) | Ischemic | 1.16 | 1.04–1.30 |
| CHARLS-Male Cohort | China, 3 724 men | Total stroke | 1.29 | 1.13–1.48 |
| Physicians’ Health Study | US male physicians (n = 21 867) | Total stroke | 1.42 | 1.18–1.70 |
| INTERSTROKE | 32 countries, 13 462 cases | Total stroke | 1.34 | 1.24–1.45 |
| Wang et al. Meta-analysis | 97 cohorts, 1.8 million | Total stroke | 1.27 | 1.20–1.33 |

**C-2 Category-Specific RRs Used in Monte Carlo**

| BMI Category | HUNT | CHARLS-M | Physicians’ | INTERSTROKE | Meta-analysis |
| --- | --- | --- | --- | --- | --- |
| <18.5 kg m⁻² | 0.92 | 1.02 | 0.95 | 0.98 | 1.01 |
| 18.5–24.9 | 1.00 (ref) | 1.00 | 1.00 | 1.00 | 1.00 |
| 25.0–29.9 | 1.13 | 1.19 | 1.28 | 1.21 | 1.18 |
| ≥30.0 | 1.31 | 1.42 | 1.56 | 1.47 | 1.36 |

**C-3 Variance–Covariance Structure**

Where studies reported category RRs without covariance, we assumed independence; otherwise, published variance–covariance matrices (INTERSTROKE) were imported directly.

**C-4 Harmonisation Steps**

- Unit Conversion: All RRs standardised to BMI per 5 kg m⁻² or categorical cut-offs.
- Outcome Mapping: Ischemic-only (HUNT) RRs were applied to total stroke under the assumption that ischemic events constitute 87% of Hungarian stroke burden (NEAK 2023).
- Sex Adjustment: CHARLS‐male estimates were scaled by 0.94 to approximate mixed-sex populations, reflecting pooled sex-differences in meta-analysis.

**C-5 Model Quality Scores (GRADE)**

## Quality Assessment Domains:

- Risk of Bias: Potential for confounding, selection bias, or measurement error within each study
- Indirectness: How well each study population generalizes to Hungarian adults
- Inconsistency: Degree of heterogeneity across included studies (for meta-analyses)
- Precision: Width of confidence intervals and statistical power

## Quality Ratings:

| Model | Risk of Bias | Indirectness | Inconsistency | Precision | Overall Quality |
| --- | --- | --- | --- | --- | --- |
| HUNT | Low | Moderate¹ | Low | High | Moderate |
| CHARLS-Male | Moderate | High² | Moderate | Moderate | Low |
| Physicians' Health | Low | Moderate³ | Moderate | Moderate | Moderate |
| INTERSTROKE | Low | Low⁴ | Low | High | High |
| Meta-analysis | Low | Low | Low | High | High |

## Footnotes:

- ¹ Norwegian population; ischemic stroke only; different healthcare system
- ² Chinese males only; non-Western diet/lifestyle patterns; single-sex limitation
- ³ US male physicians; highly selected/educated cohort; occupational homogeneity
- ⁴ 32-country case-control; includes Central-Eastern European populations

## Interpretation:

INTERSTROKE and the Wang et al. meta-analysis received **"High certainty"** ratings due to their global scope and inclusion of diverse populations, including Central and Eastern Europe. CHARLS-Male received **"Low certainty"** for Hungarian application due to geographic and sex-specific limitations.

We retained all five models to provide a comprehensive range of estimates (conservative to optimistic scenarios) rather than relying on a single source, acknowledging uncertainty in model transferability.

**Appendix D: Regional Comparison of Overweight and Obesity Prevalence**

**D-1 Regional Comparison of Overweight and Obesity Prevalence Based on EUROSTAT and Current Study Data**

| Country/Region | Overweight + Obesity (%) | Year | Source | Rank |
| --- | --- | --- | --- | --- |
| Hungary (this study) | 72.5 | 2022 | Own data | 1 |
| Malta | 72.1 | 2019 | EUROSTAT | 2 |
| Hungary (EHIS) | 64.1 | 2019 | EUROSTAT | 3 |
| Slovakia | 59.2 | 2019 | EUROSTAT | 4 |
| Czech Republic | 56.8 | 2019 | EUROSTAT | 5 |
| Romania | 54.7 | 2019 | EUROSTAT | 6 |
| EU average | 51.6 | 2019 | EUROSTAT | – |
| Austria | 46.3 | 2019 | EUROSTAT | 8 |

**Note:**

Hungary's 2022 prevalence (72.5%) represents an 8.4 percentage point increase from the 2019 EHIS estimate (64.1%), exceeding all Central and Eastern European countries. The 20.9 percentage point excess above the EU average (51.6%) highlights the urgent need for targeted obesity prevention interventions. This 13-18 percentage point gap with neighboring countries (Slovakia, Romania) suggests that Hungary faces unique challenges, potentially related to specific dietary customs, socioeconomic transitions, and healthcare system constraints characteristic of post-socialist countries.
